# Tanshinone IIA Inhibits Hypoxia-Induced Pulmonary Artery Smooth Muscle Cell Proliferation via Akt/Skp2/p27-Associated Pathway

**DOI:** 10.1371/journal.pone.0056774

**Published:** 2013-02-21

**Authors:** Ying Luo, Dun-Quan Xu, Hai-Ying Dong, Bo Zhang, Yi Liu, Wen Niu, Ming-Qing Dong, Zhi-Chao Li

**Affiliations:** 1 Department of Pathology, Xijing Hospital, Fourth Military Medical University, Xi’an, People’s Republic of China; 2 Department of Pathology and Pathophysiology, Fourth Military Medical University, Xìan, People’s Republic of China; Vanderbilt University Medical Center, United States of America

## Abstract

We previously showed that tanshinone IIA ameliorated the hypoxia-induced pulmonary hypertension (HPH) partially by attenuating pulmonary artery remodeling. The hypoxia-induced proliferation of pulmonary artery smooth muscle cells (PASMCs) is one of the major causes for pulmonary arterial remodeling, therefore the present study was performed to explore the effects and underlying mechanism of tanshinone IIA on the hypoxia-induced PASMCs proliferation. PASMCs were isolated from male Sprague-Dawley rats and cultured in normoxic (21%) or hypoxic (3%) condition. Cell proliferation was measured with 3 - (4, 5 - dimethylthiazal - 2 - yl) - 2, 5 - diphenyltetrazoliumbromide assay and cell counting. Cell cycle was measured with flow cytometry. The expression of of p27, Skp-2 and the phosphorylation of Akt were measured using western blot and/or RT-PCR respectively. The results showed that tanshinone IIA significantly inhibited the hypoxia-induced PASMCs proliferation in a concentration-dependent manner and arrested the cells in G1/G0-phase. Tanshinone IIA reversed the hypoxia-induced reduction of p27 protein, a cyclin-dependent kinase inhibitor, in PASMCs by slowing down its degradation. Knockdown of p27 with specific siRNA abolished the anti-proliferation of tanshinone IIA. Moreover, tanshinone IIA inhibited the hypoxia-induced increase of S-phase kinase-associated protein 2 (Skp2) and the phosphorylation of Akt, both of which are involved in the degradation of p27 protein. In vivo tanshinone IIA significantly upregulated the hypoxia-induced p27 protein reduction and downregulated the hypoxia-induced Skp2 increase in pulmonary arteries in HPH rats. Therefore, we propose that the inhibition of tanshinone IIA on hypoxia-induce PASMCs proliferation may be due to arresting the cells in G1/G0-phase by slowing down the hypoxia-induced degradation of p27 via Akt/Skp2-associated pathway. The novel information partially explained the anti-remodeling property of tanshinone IIA on pulmonary artery in HPH.

## Introduction

Chronic hypoxia-induced pulmonary hypertension (HPH), characterized by the sustained pulmonary artery constriction and progressive structure remodeling, contributes to the morbidity and mortality of adult and pediatric patients with various lung and heart diseases [1]. Pulmonary artery structure remodeling plays a key role in the persistent deterioration and the difficult reverse of HPH [1]. Current therapies mainly focus on altering the vasoconstrictive elements. There are few effective therapies to halt the progression of HPH [2]. One of the major causes for chronic hypoxia-induced pulmonary arterial remodeling is the aberrant proliferation of pulmonary artery smooth muscle cells (PASMCs) [3]. So inhibition of the aberrant proliferation of PASMCs may halt the deteriorative progress of HPH.

The balance of cell proliferation and quiescence is delicately regulated by cyclin - dependent kinases (CDKs) and CDK inhibitors [4]. As one of the important CDK inhibitors, p27 (p27kip1) inhibits G1 cyclin/CDK complexes and blocks the G1-S transition in the cell cycle [5]. Previous studies have shown that p27 was the only CDK inhibitor decreased in the lung in HPH mice and played an important role in regulating PASMC proliferation [4], [6]. The protein level of p27 is mainly regulated at the degradation step [7], [8]. The S-phase kinase-associated protein 2 (Skp2) functions specifically for the proteolytic degradation of p27 protein [9].

Tanshinone IIA is one of the main active components and representative in Danshen, the dried root of Salvia Miltiorrhiza, and widely used in Asian countries especially in China for the treatment of various cardiovascular diseases [10], [11]. Previously we showed that tanshinone IIA decreased pulmonary artery pressure and ameliorated the remodeling of pulmonary artery in HPH rats [12]. Tanshinone IIA reversed the sustained vasoconstriction of remodeled pulmonary arteries from HPH rats by inhibiting extracellular Ca^2+^ influx and intracellular Ca^2+^ release in vitro [13]. However, why tanshinone IIA ameliorated the hypoxia-induced remodeling of pulmonary artery is not full defined.

Considering the key role of the hypoxia-induced aberrant proliferation of PASMCs in the pulmonary arterial remodeling, the present study was designed to investigate the effects and the underlying mechanism of tanshinone IIA on PASMCs proliferation, especially by elucidating its action on cell cycle and cyclin-dependent kinase inhibitor p27 pathway. We found that in vitro tanshinone IIA inhibited the hypoxia-induced PASMCs proliferation, arrested PASMCs in G1/G0-phase and slowed down the degradation of p27 protein in PASMCs via decreasing the phosphorylation of Akt and the production of Skp2. Furthermore, tanshinone IIA significantly upregulated the hypoxia-induced p27 protein reduction and downregulated the hypoxia-induced Skp2 increase in pulmonary arteries in HPH rats. The present study firstly demonstrated that tanshinone IIA modulated the hypoxia-induced PASMC proliferation via Akt/Skp2/p27-associated pathway. The novel information partially explained the anti-remodeling property of tanshinone IIA on pulmonary artery in HPH rats.

## Materials and Methods

### Ethics Statement

All the experiments were approved by the Animal Care and Use Committee of the Fourth Military Medical University and complied with the Declaration of the National Institutes of Health Guide for Care and Use of Laboratory Animals.

### Reagents

Tanshinone IIA (sodium sulphonate, purity is 99.0%), purchased from National Institute for the Control of Pharmaceutical and Biological Products (Beijing, China), was prepared as 5 mg/mL stock solution in distilled water and kept in dark at 4°C. The following antibodies were used for western blot: p27 monoclonal antibody (1∶1000; Millipore, Bedford, USA), Skp-2 polyclonal antibody (1∶50; Abcam, Cambridge, UK), smooth muscle alpha-actin and smooth muscle-myosin heavy chain antibody (1∶5000; Sigma, St. Louis, MO, USA), beta-actin antibody (1∶10000; Sigma, St.Louis, MO, USA), Akt and phospho-Akt (1∶1000; Cell Signaling, Massachusetts, USA). Other chemicals were from Sigma, and the BCA protein assay kit and ECL kit were from Pierce, USA.

### Cell Culture

Male Sprague - Dawley rats (200–220 g) were anesthetized and the pulmonary arteries were removed rapidly. PASMCs were cultured by tissue explant method [12] and grown in RPMI 1640 medium supplemented with 20% fetal bovine serum (Gibco) in 5% CO2 and 95% air at 37°C. At confluence, the cells were trypsinized, split in a 1∶3 ratio, and recultured in RPMI 1640 supplemented with 5% FBS. The 3th to 5th generation cells were used for the subsequent experiments. Smooth muscle cell identity was verified by positive staining for smooth muscle alpha-actin and smooth muscle-myosin heavy chain at each passage (>95% of cells stained positive for the two markers).

### Cell Viability Assay

Cell viability was measured by trypan blue exclusion [12]. Briefly, PASMCs were seeded in 24 - well plates at a density of 5×10^4^ cells each well. The cells were then cultured in RPMI 1640 supplemented with 5% FBS plus diverse dose of tanshinone IIA under normoxic (21% O_2_) or hypoxic condition (3% O_2_) (the same for the following experiments) for 24 h, and immediately assessed for viability by trypan blue exclusion.

### Cell Proliferation Assay

PASMCs proliferation was measured by 3 - (4, 5 - dimethylthiazal - 2 - yl) - 2, 5 - diphenyltetrazoliumbromide (MTT) assay and cell counting as previously described [12]. For MTT assay, PASMCs were seeded in 96-well plates at a density of 5,000 cells each well. After serum starved for 48 h in serum-free medium, the cells were then cultured in RPMI 1640 containing 5% FBS plus diverse dose of tanshinone IIA (0, 3, 10, 30 and 50 µg/mL) for 24 h under normoxic or hypoxic condition. At the end of treatment, 10 µL MTT (5 mg/mL)/well was added to the plates, and incubated for another 4 h at 37°C. The supernatant was then carefully removed, and 75 µL/well dimethyl sulfoxide (DMSO) was added to dissolve the formazan crystals. The absorbance of the solubilized product at 490 nm (A490) was measured with microplate spectrophotometer (PowerWave XS, BioTek Inc, Vermont, USA).

For cell counting, PASMCs were seeded in 24-well plates at a density of 5×10^4^ cells/well, and then were treated with the above conditions. At the end of treatment, they were washed with phosphate buffered solution, harvested by mild trypsinization, and counted with a hematocytometer.

### Cell Treatment

PASMCs were seeded in RPMI 1640 containing 5% FBS at a density of 1×10^6^ cells/well in 6-well plate. After being serum-starved for 48 h (0.1% serum), PASMCs were cultured in RPMI 1640 supplemented with 5% FBS plus different concentration of tanshinone IIA for 24 h under normoxic or hypoxic condition. Then the cells were collected for cell cycle analysis or extraction of RNA and protein.

### Cell Cycle Analysis

Cell cycle was determined by flow cytometry. Cells were harvested with 0.25 g/L trypsin from 6-well plates, resuspended in 10 µL PBS, added in 1 mL 70% ethanol, centrifuged and washed with cold PBS. Then cells were resuspended in PBS with 20 µg/mL PI and 1 mg/mL RNAse. After incubating for 15 minutes at room temperature, the samples were run on a 7 Laser SORP BD LSR II system and data was collected with DIVA software on LSR II and analyzed with FlowJo v8.8.6.

### Western Blot Analysis

The total lysate was obtained from harvested pulmonary arteries or cultured PASMCs. The protein was extracted according to the instructions of Total Protein Extraction Kit. The protein concentration was determined with BCA protein assay kit. The samples were separated on denaturing 10% SDS-polyacrylamide gel and transferred to a nitrocellulose membrane. The membrane was blocked with Tris buffered saline containing 5% non - fat dry milk at room temperature for 2 h, followed by incubation with primary antibody against beta-actin, p27, Skp2, total-Akt or phospho-Akt overnight at 4°C. Then secondary antibody (anti-mouse IgG peroxidase conjugated, 1∶ 5000) was incubated. The signal detection was performed by using the enhanced chemiluminescence system (Amersham, Arlington Heights, IL, USA) of a commercial ECL kit.

### RNA Extraction and Reverse-transcription Polymerase Chain Reaction (RT-PCR)

The total mRNA was extracted from harvested pulmonary arteries or cultured PASMCs using Trizol reagent. Total RNA (2 µg) was used to carry out RT-PCR to measure mRNA expression with Qiagen Onestep RT-PCR Kit (Qiagen) according to the manual. Cycling conditions were as follows: 95°C for 5 minutes followed by 35 cycles of amplification (95°C denaturation for 30 seconds, annealing for 30 seconds (51°C for p27, 59°C for Skp2 and 55°C for beta-actin), 72°C extension for 40 seconds). The primer pairs for p27 (amplicon size: 319 bp) were (forward) 5′-CTTGGAGAAGCACTGCCGAGAT-3′ and (reverse) 5′-CCCTGGACACTGCTCCGCTA-3′, for Skp-2 (amplicon size: 396 bp) were (forward) 5′-TAAGCGTTAGGTCTTTGGAA-3′ and (reverse) 5′-TGGTTGTGTGTGTCTGTGTC3’, and for beta-actin (amplicon size: 270 bp) were (forward) 5′-ATCATGTTTGAGACCTTCAACA-3′ and (reverse) 5′-CATCTCTTGCTCGAAGTCCA-3′ respectively. PCR products were run on 1% agarose gel and stained with EB to ensure the expected molecular size amplicons.

### SiRNA Transfection

PASMCs were seeded at a density of 1×10^6^ cells/well in a 6-well plate and cultured in nomoxia. The cells were divided into six groups: hypoxia group, hypoxia plus tanshinone IIA group, hypoxia plus scramble siRNA group, hypoxia plus scramble siRNA and tanshinone IIA group, hypoxia plus siRNA group, and hypoxia plus siRNA and tanshinone IIA group. The next day the cells were transfected with siRNA accordingly. Six hours later, the medium was changed and tanshinone IIA (10 µg/mL) or the same volume of phosphate buffer saline were added according to the groups. Then the cells were cultured in hypoxia (3%) for another 24 h, and then applied to MTT assay or western blot. A special siRNA sequence for p27 was designed according to previously published work [14]. The transfection was performed using Fugene HD transfection reagent (Roche).

### Degradation Assay

To assess the effects of tanshinone IIA on the degradation of p27 in PASMCs, cells were seeded in a 6-plate at density of 1×10^6^ cells/well and cultured for 24 h, then cycloheximide (50 µg/mL) or cycloheximide plus tanshinone IIA (10 µg/mL) was added to the medium. Cells were collected at 0 h, 2 h, 4 h, 6 h after the drug was added. The total protein was extracted and P27 protein at different time point was quantified by western blot. The quantification was processed by using densitometric analysis. The protein of p27 at different time point was normalized by dividing the protein level at 0 h. The normalized data were fitted with linear curve and t_1/2_ (half-life) of p27 protein was measured as the time when 50% of p27 protein degraded.

### Animal Model

Male Sprague-Dawley rats (200–220 g) were randomly divided into four groups (all n = 8): normoxia, normoxia plus tanshinone IIA, hypoxia, and hypoxia plus tanshinone IIA. Tanshinone IIA was dissolved in distilled water. The same volume of physiological saline was used as the vehicle. Rats were maintained in room air (for normoxia group and normoxia plus tanshinone IIA group) or in a specially designed hypobaric chamber depressurized to 380 mmHg (oxygen concentration reduced to about 10%, for hypoxia group and hypoxia plus tanshinone IIA group) for four weeks. Tanshinone IIA (10 mg/kg/day, for normoxia plus tanshinone IIA group and hypoxia plus tanshinone IIA group) or vehicle (for normoxia group and hypoxia group) was intraperitoneally injected every day. All rats were maintained in a 12 to 12 hours light-dark cycle in an air-conditioned room (25°C) with a relative humidity of 65% and had free access to food and water.

After four weeks exposure of hypoxia, the rats in every group were anesthetized with 20% ethylurethanm (4 mL/kg, i.p.). The measurement of right ventricular pressure and the hematoxylin and eosin staining of small part of lung tissue were done. The increased right ventricular pressure and thickened tunica media in small distal pulmonary arteries confirmed the successful establishment of hypoxic pulmonary hypertension model (data not shown). The rest parts of the lungs were used to isolate the pulmonary artery (to the third division) under a dissecting microscope. The obtained pulmonary arteries were used to extract total RNA and protein and carry out RT-PCR and western blot.

### Statistical Analyses

All values were expressed as mean ± SEM. The statistical significance of differences between groups was evaluated by one-way analysis of variance (ANOVA), followed by Dunnett’s test for multiple comparisons (SPSS for Windows version 16.0, Chicago, USA). P<0.05 was considered as statistically significant.

## Results

### Effects of Tanshinone IIA on PASMCs Proliferation under Normoxic and Hypoxic Conditions

**Figure 1:**
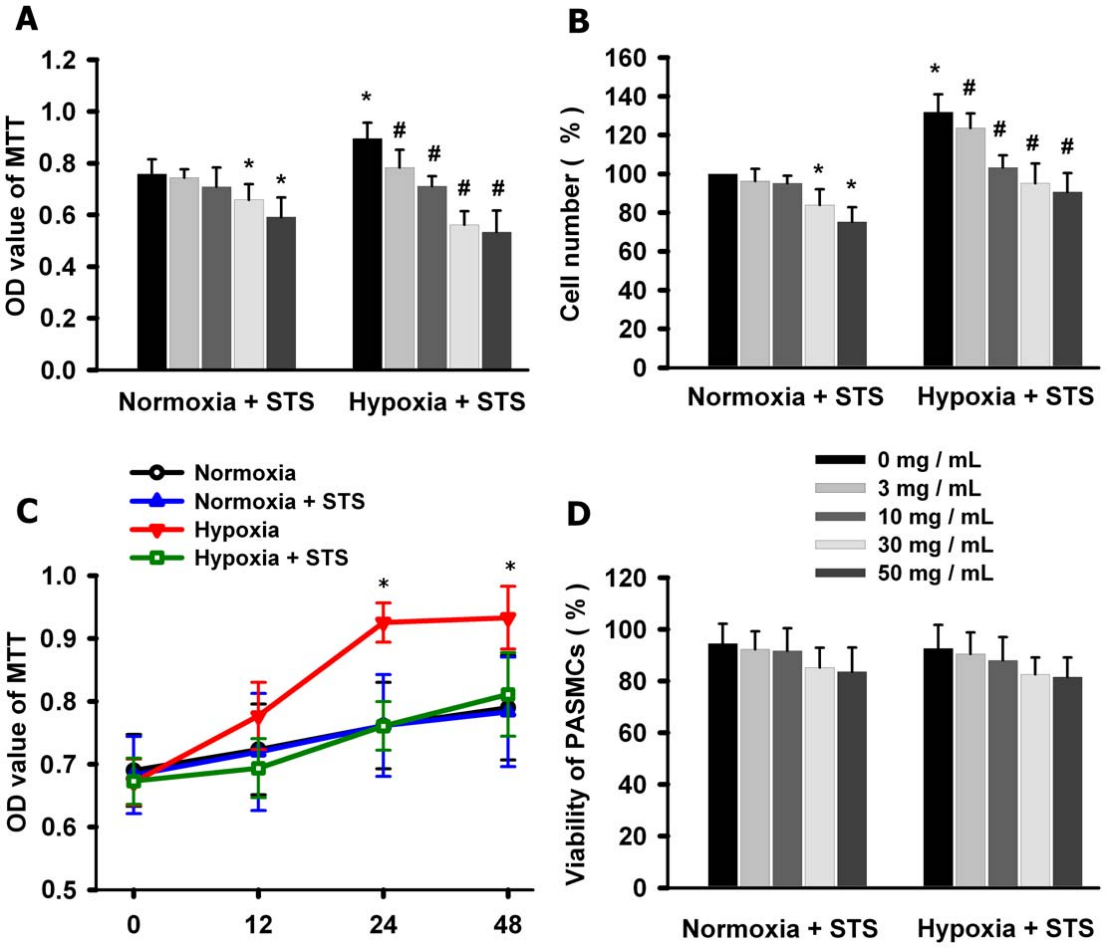
Effects of tanshinone IIA on PASMCs proliferation under normoxic and hypoxic conditions. (A–B) Effects of tanshinone IIA on the proliferation of PASMCs under normoxic (21% O_2_) and hypoxic (3% O_2_) conditions. Hypoxia for 24 h significantly promoted PASMCs proliferation. Tanshinone IIA significantly inhibited hypoxia-induced PASMCs proliferation. At 3 and 10 µg/mL, tanshinone IIA only inhibited PASMCs proliferation under hypoxic condition, but at 30 and 50 µg/mL Tanshinone IIA inhibited PASMCs proliferation under both hypoxic and normoxic conditions (*P<0.05 vs normoxic plus 0 µg/mL tanshinone IIA group, *^#^P*<0.05 vs hypoxia plus 0 µg/mL tanshinone IIA group). (C) Time course experiments showed that hypoxia for 24 h reached maxmal stimulation on PASMCs proliferation and tanshinone IIA reached maximal inhibition after 24 h incubation (*P<0.05 vs corresponding time points in hypoxia group). (D) Effect of tanshinone IIA on PASMCs viability. Tanshinone IIA (3–50 µg/mL) had no significant effects on PASMCs viability under both normoxic and hypoxic conditions. Data are mean ± SEM from three replicate experiments. In this and the following figures, STS represents tanshinone IIA (sodium sulphonate).

Firstly we evaluated the effects of tanshinone IIA on PASMCs proliferation under normoxic (21% O_2_) and hypoxic (3% O_2_) conditions. MTT assay (figure 1 A) showed that hypoxia for 24 h significantly promoted PASMCs proliferation. Tanshinone IIA inhibited the hypoxia-induced PASMCs proliferation in a concentration-dependent manner. At 3 and 10 µg/mL, tanshinone IIA only inhibited PASMCs proliferation under hypoxic condition, but at 30 and 50 µg/mL Tanshinone IIA inhibited PASMCs proliferation under both hypoxic and normoxic conditions. The cell counting assay showed the similar results (figure 1 B). The time course experiments showed that hypoxia for 24 h reached maximal stimulation on PASMCs proliferation and tanshinone IIA reached maximal inhibition after 24 h incubation (figure 1 C). The cell viability experiments showed that tanshinone IIA at 3, 10, 30 and 50 µg/mL had no significant cytotoxicity on PASMCs under both normoxic and hypoxic conditions (figure 1 D). Tanshinone IIA at 10 µg/mL almost reversed the hypoxia-induced proliferation of PASMCs. Therefore, the concentration of 10 µg/mL was used in the following experiments.

### Tanshinone IIA Arrested PASMCs in G1/G0-phase under Hypoxic Condition

**Figure 2:**
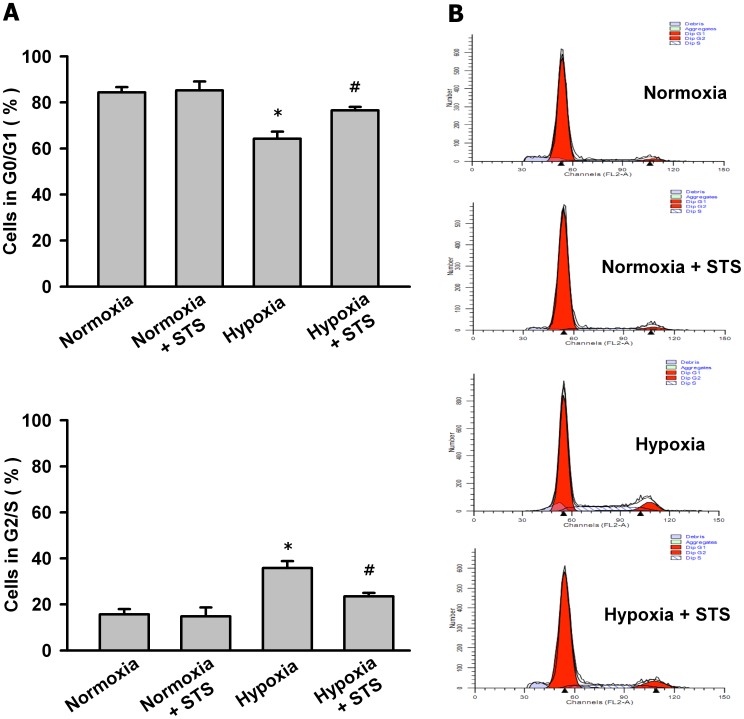
Tanshinone IIA arrested PASMCs in G1/G0-phase under hypoxic condition. (A) Effects of tanshinone IIA on the cell cycle of PASMCs under normoxic (21% O_2_) and hypoxic (3% O_2_) conditions. Hypoxia for 24 h decreased PASMCs in G0/G1-phase and increased PASMCs in G2/S-phase. Tanshinone IIA (10 µg/mL) reversed the effects of hypoxia and arrested more PASMCs in G1/G0-phase. *P<0.05 vs normoxia group. *^#^P*<0.05 vs hypoxia group. Data are mean ± SEM from three replicate experiments. (B) Representative cell cycle histograms measured by flowcytometry.

Cell proliferation depends on the cell cycle transition from G1/G0- to G2/S-phase. Then we determined whether tanshinone IIA affected the cell cycle of PASMCs under hypoxic condition. As shown in figure 2, hypoxia for 24 h promoted PASMCs to enter the cell mitosis cycle, decreased PASMCs in G0/G1-phase and increased PASMCs in G2/S-phase. Tanshinone IIA at 10 µg/mL reversed the effects of hypoxia on PASMC cell cycle as it arrested more PASMCs in G1/G0-phase, increased PASMCs G0/G1-phase and decreased PASMCs in G2/S-phase.

### Tanshinone IIA Increased p27 Protein in PASMCs under Hypoxic Condition

**Figure 3:**
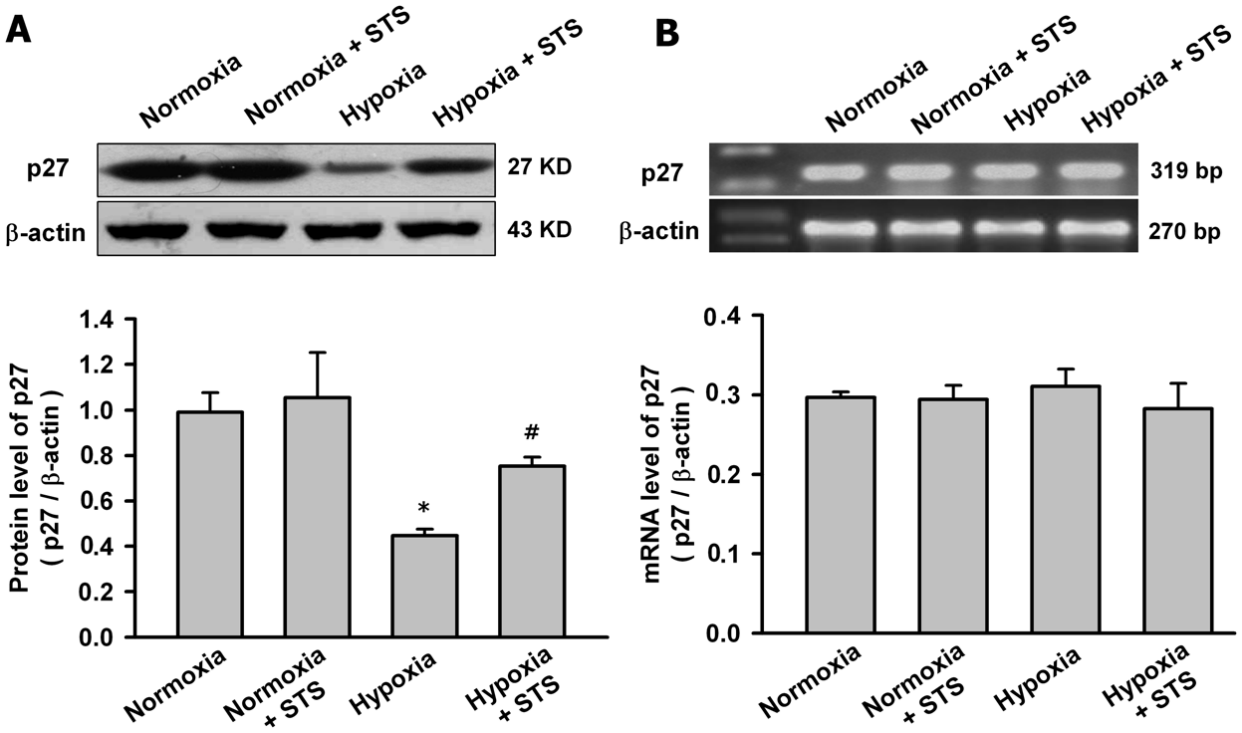
Tanshinone IIA upregulated the protein level of p27 in PASMCs under hypoxic condition. Effects of tanshinone IIA on p27 protein (A) and mRNA (B) in PASMCs under normoxic (21% O_2_) or hypoxic (3% O_2_) condition. Beta-actin was used as control. Hypoxia for 24 h significantly decreased the protein level but not the mRNA level of p27in PASMCs (*P<0.05 vs normoxia group, mean ± SEM, n = 3). Tanshinone IIA at 10 µg/mL had no significant effects on the mRNA level of p27 in PASMCs under both hypoxic and normoxic condition, but significantly upregulated the protein level of p27 under hypoxic condition (*^#^P*<0.05 vs hypoxia group, mean ± SEM, n = 3).

Previous studies showed that p27 decreased in the lung in HPH mice and played a key role in PASMC proliferation and G1 to S-phase transition in cell cycle [4], [6], so next we evaluated the effects of hypoxia and tanshinone IIA on p27 in PASMC. Figure 3 showed that hypoxia for 24 h significantly decreased p27 protein but not mRNA in PASMCs. Tanshinone IIA had no significant effects on p27 mRNA in PASMCs under both hypoxic and normoxic conditions but only significantly increased p27 protein under hypoxic condition.

### Knockdown of p27 Abolished the Inhibition of Tanshinone IIA on the Hypoxia-induced PASMCs Proliferation

**Figure 4:**
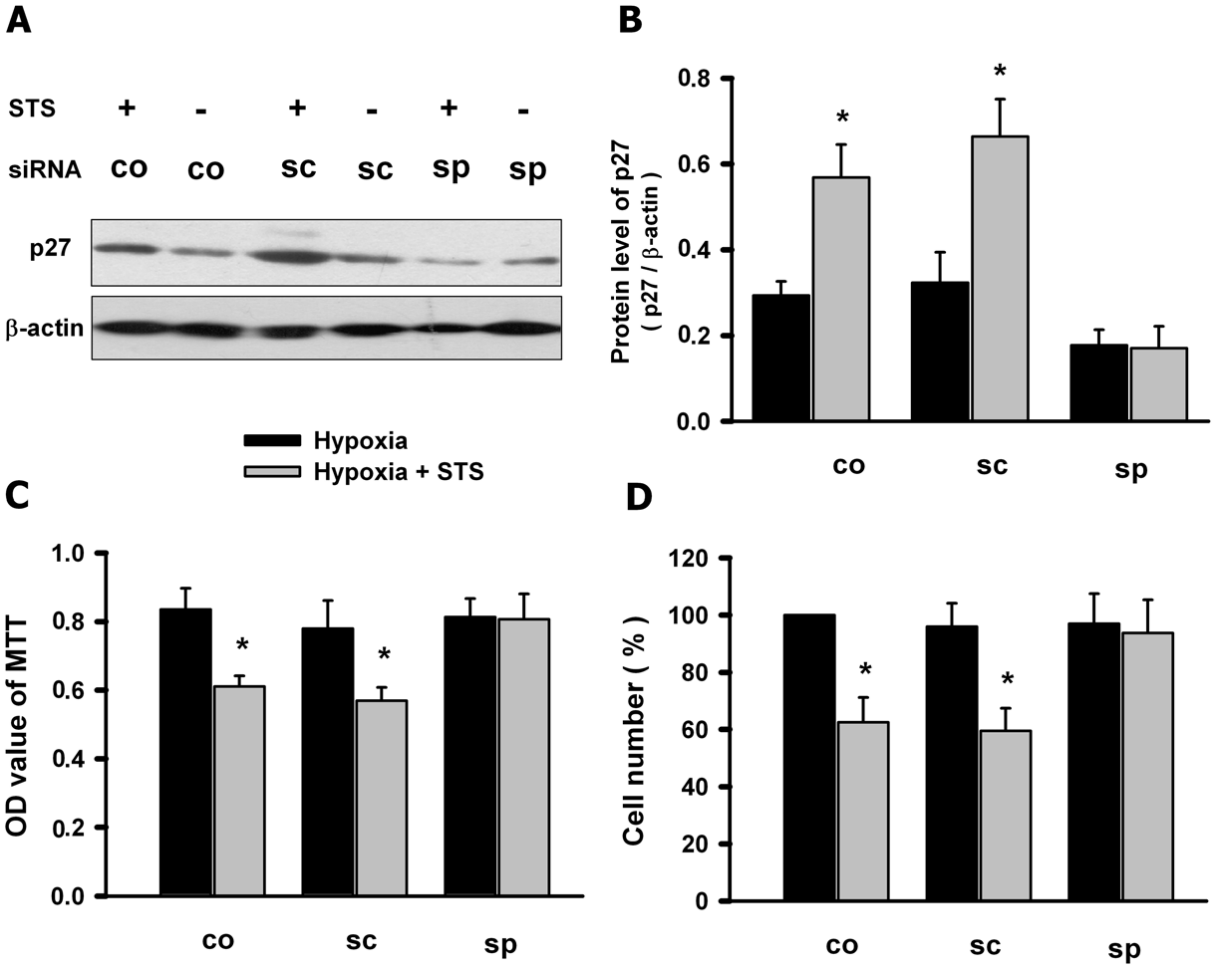
Knockdown of p27 abolished the inhibition of tanshinone IIA on hypoxia-induced PASMCs proliferation. (A) Representative western blot for p27 protein in PASMCs transfected with specific siRNA for p27 (sp) or scramble sequence (sc) or control group (co) under hypoxic (3% O_2_ for 24 h) condition. Summarized data for three experiments was shown in panel (B). Beta-actin was used as control. Specific siRNA for p27 (sp) significantly reversed tanshinone IIA-induced increase of p27 protein in PASMCs under hypoxic condition Scrambled sequence (sc) had no significant effects on p27 protein. (C–D) MTT assay and cell counting showed that specific siRNA for p27 (sp) but not the scrambled sequence (sc) abolished the inhibition of tanshinone IIA on the hypoxia-induced PASMCs proliferation. *p<0.05 vs corresponding hypoxia group. Data are mean ± SEM from three replicate experiments.

Then we determined whether knockdown of p27 affected the inhibition of tanshinone IIA on the hypoxia-induced PASMCs proliferation. PASMCs were transfected with specific siRNA for p27 or scrambled sequence, then treated with tanshinone IIA under hypoxic condition. Figure 4 A showed that specific siRNA for p27 significantly reversed tanshinone IIA-induced increase of p27 protein in PASMCs under hypoxic condition. MTT assay and cell counting showed that the knockdown of p27 with specific siRNA almost abolished the inhibition effect of tanshinone IIA on the hypoxia-induced PASMCs proliferation. The scrambled sequence had no significant effects on p27 protein and the inhibition of tanshinone IIA on the hypoxia-induced PASMCs proliferation (figure 4. B). These data suggested that the p27 was necessary for the tanshinone IIA-induced inhibition of PASMCs proliferation.

### Tanshinone IIA Slowed Down the Hypoxia-induced Degradation of p27 Protein in PASMCs under Hypoxic Condition

**Figure 5:**
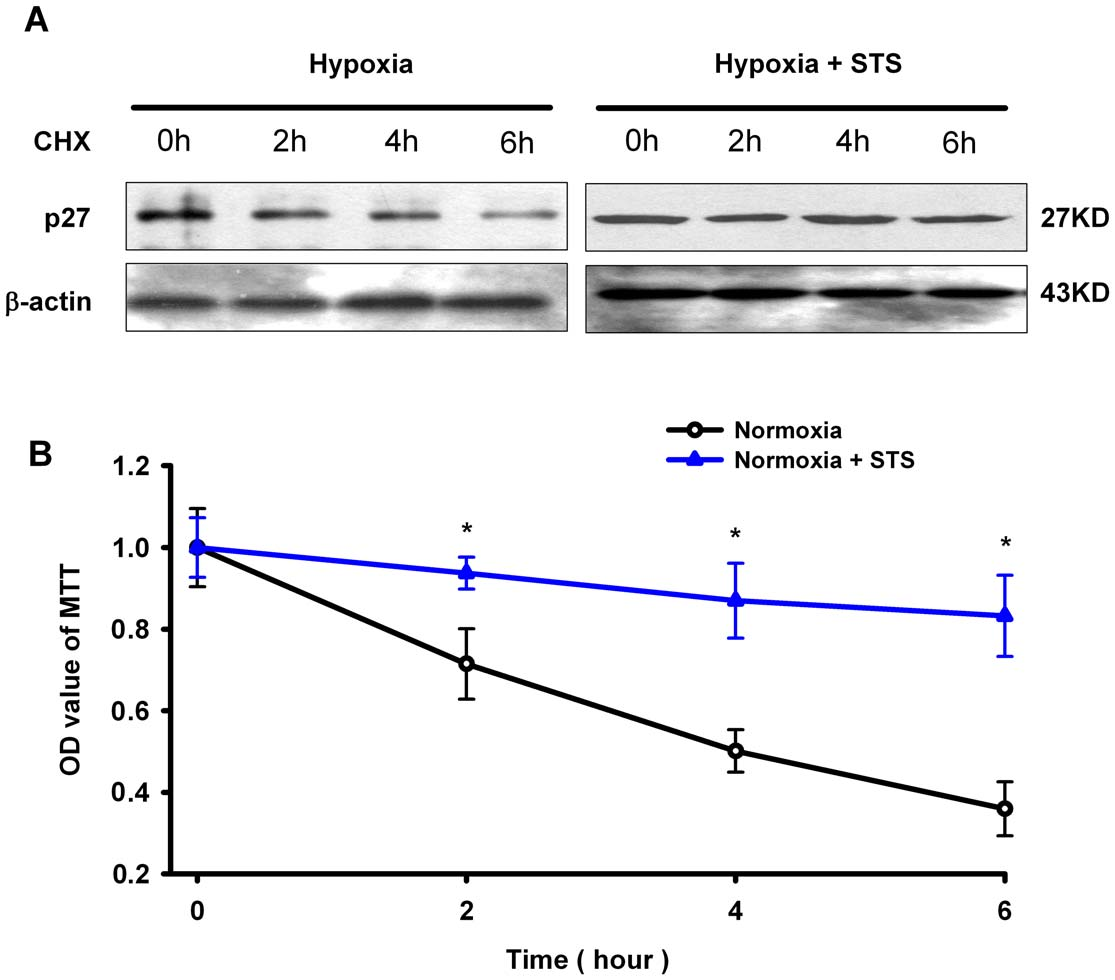
Tanshinone IIA slowed down the degradation of p27 protein in PASMCs under hypoxic condition. (A) Representative western blot for p27 protein in PASMCs at different time point after cycloheximide (50 µg/mL) added under hypoxia (3%) or hypoxia plus tanshinone IIA (10 µg/mL) condition. Beta-actin was used as control. Summarized data from three experments were shown panel (B). Data were normalized by dividing the protein level at time zero. P27 protein degraded fast but tanshinone IIA significantly slowed down the degradation of p27 protein (*P<0.05 vs corresponding time point in the same group. mean ± SEM, n = 3). CHX represents cycloheximide (50 µg/mL).

To explore whether tanshinone IIA modulated the degradation of p27 protein in PASMCs under hypoxic condition, we measured the effects of tanshinone IIA on the half-time (t_1/2_) of p27 protein in PASMCs. As shown in figure 5, p27 protein in PASMCs degraded faster under hypoxia than under hypoxia plus tanshinone IIA condition. The fitted t_1/2_ of p27 protein was 4.2 h in hypoxia and 15.6 h in hypoxia plus tanshinone IIA condition respectively. Tanshinone IIA at 10 µg/mL significantly slowed down the degradation and prolonged the half-life of p27 protein in PASMCs under hypoxic condition.

### Tanshinone IIA Inhibited the Hypoxia-induced Increase of Skp2 and the Phosphorylation of Akt in PASMCs

**Figure 6:**
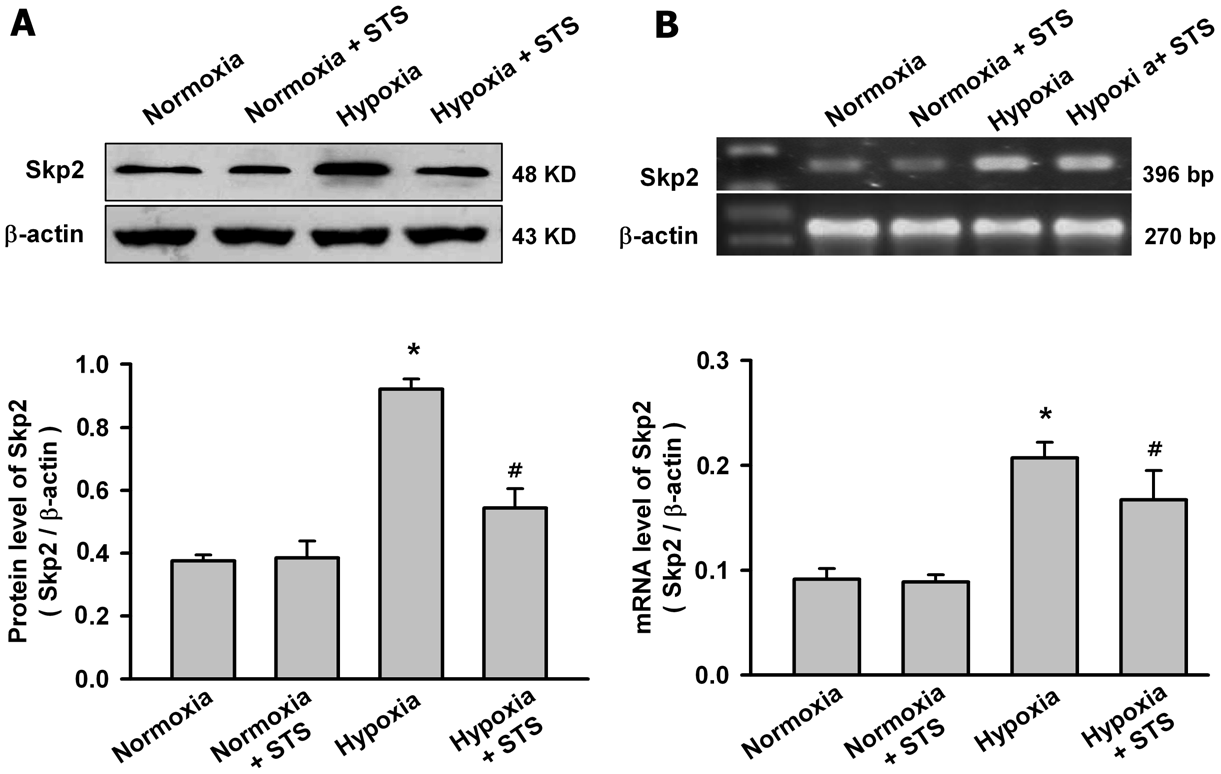
Tanshinone IIA inhibited the increase of Skp2 expression in PASMCs under hypoxic condition. Effects of tanshinone IIA on the protein (A) and mRNA (B) level of Skp2 in PASMCs under normoxic (21% O_2_) or hypoxic (3% O_2_) condition. Representative western blot for Skp2 protein and agarose gel electrophoresis for RT-PCR products of Skp2 mRNA from different groups are shown in the top of the panel. Beta-actin was used as control. Summarized data are shown in the bottom of the panel. Hypoxia for 24 h significantly increased the Skp2 protein and mRNA level in PASMCs (*P<0.05 vs normoxia group, mean ± SEM, n = 3). Tanshinone IIA at 10 µg/mL significantly reduced hypoxia-induced increase of Skp2 in both mRNA and protein levels, but had no significant effects on the Skp2 expression in PASMCs under normoxic condition (*^#^P*<0.05 vs hypoxia group, mean ± SEM, n = 3).

Skp2 is a rate-limiting component of the machinery that ubiquitinates and degrades p27 protein, whereas phosphorylation of Akt is the key step for Skp2 expression. So we evaluated whether tanshinone IIA decreased the degradation of p27 via regulating Skp2 and the phosphorylation of Akt. As shown in figure 6, hypoxia for 24 h significantly increased the mRNA and protein level of Skp2 in PASMCs. Tanshinone IIA at 10 µg/mL significantly reduced the hypoxia-induced increase of the mRNA and protein level of Skp2, but had no significantly effects on the Skp2 expression in PASMCs under normoxic condition.

**Figure 7:**
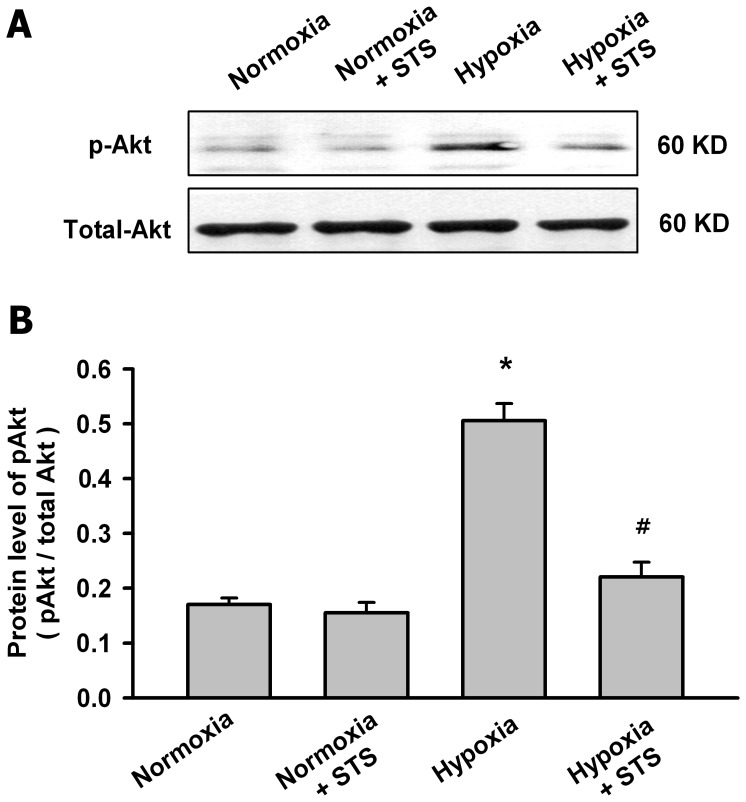
Tanshinone IIA inhibited the hypoxia-induced phosphorylation of Akt in PASMCs. (A) Representative western blot for the phosphorylated Akt and total Akt protein under normoxic or hypoxic (3% O_2_ for 24 h) condition. (B) Summarized data of the phosphorylated Akt protein. Data were normalized by dividing the total Akt protein. Hypoxia significantly increased the phosphorylation of Akt in PASMCs, but had no significant effects on the total Akt. Tanshinone IIA significantly inhibited the hypoxia-induced phosphorylation of Akt. (*P<0.05 vs normoxia group. *^#^P*<0.05 vs hypoxia group, mean ± SEM, n = 3).

Figure 7 showed that hypoxia for 24 h significantly increased the phosphorylation of Akt in PASMCs but had no significant effects on the total Akt. Tanshinone IIA at 10 µg/mL significantly inhibited the hypoxia-induced phosphorylation of Akt.

### Tanshinone IIA Regulated Skp2/p27 Axis in Pulmonary Arteries in HPH Rats

**Figure 8:**
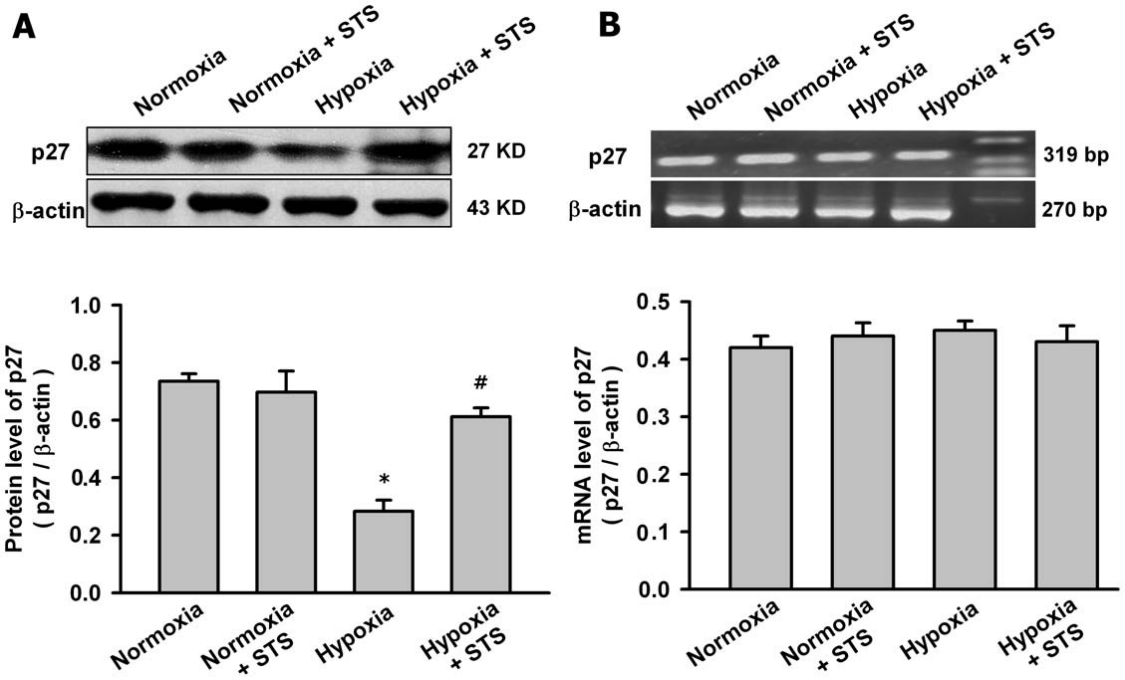
Effects of Tanshinone IIA on the expression of p27 in pulmonary arteries in HPH rats. Representative western blot for p27 protein (A) and agarose gel electrophoresis for RT-PCR products of p27 mRNA (B) in pulmonary arteries from control and HPH rats. Beta-actin was used as control. Summarized data are shown in the bottom respectively. Hypoxia significantly decreased p27 protein level in pulmonary arteries and tanshinone IIA (10 mg/kg/day^)^ significantly reversed the hypoxia-induced p27 protein reduction (*P<0.05 vs normoxia group, # P<0.05 vs hypoxia group, mean ± SEM, n = 3). Hypoxia and tanshinone IIA did not significantly affect p27 mRNA level in pulmonary arteries in HPH rats.

**Figure 9:**
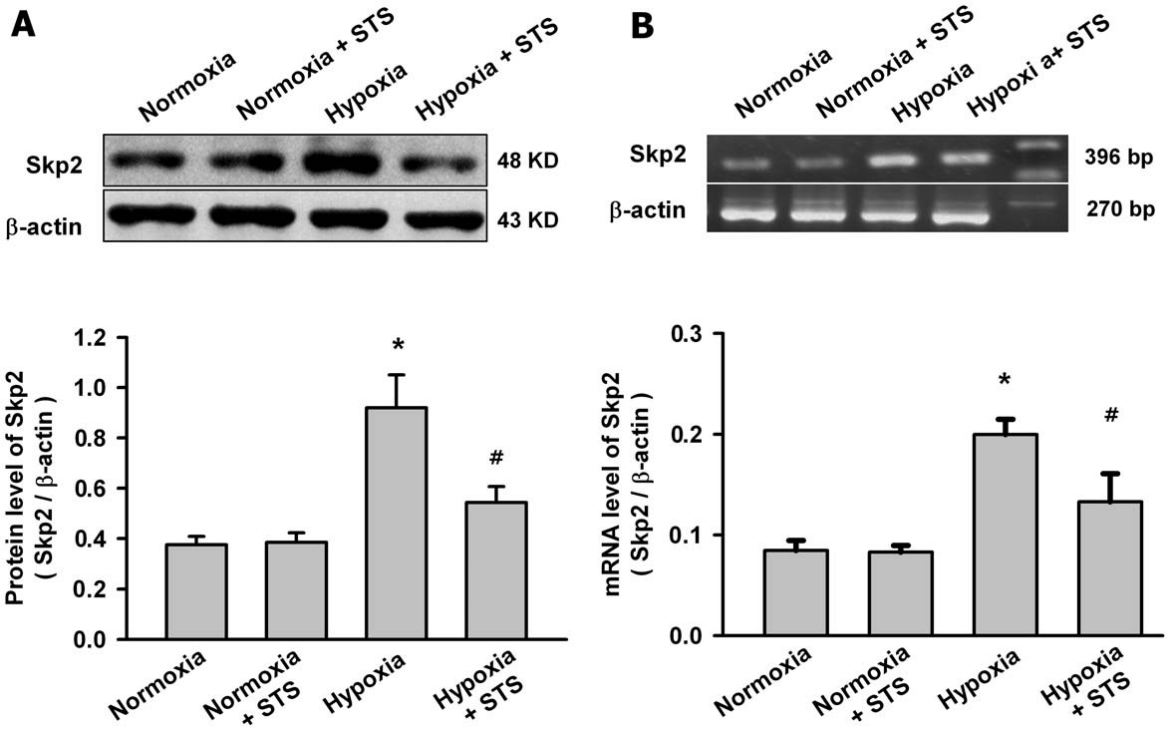
Effects of Tanshinone IIA on the expression of Skp2 in pulmonary arteries in HPH rats. Representative western blot for Skp2 protein (A) and agarose gel electrophoresis for RT-PCR products of p27 mRNA (B) in pulmonary arteries from control and HPH rats. Beta-actin was used as control. Summarized data are shown in the bottom respectively. Hypoxia significantly increased the Skp2 protein and mRNA level in pulmonary arteries from HPH rats, tanshinone IIA significantly reversed the effects of hypoxia on the expression of Skp2 (*P<0.05 vs nomoxia group, # P<0.05 vs hypoxia group, mean ± SEM, n = 3).

Then we explored whether tanshinone II could regulate Skp2/p27 axis in vivo. The protein and mRNA level of p27 and Skp2 in pulmonary arteries from HPH rats was measured. Figure 8 showed that hypoxia for four weeks significantly decreased p27 protein and tanshinone IIA (10 mg/kg/day^)^ significantly reversed the hypoxia-induced p27 protein reduction in pulmonary arteries in HPH rats. However, hypoxia and tanshinone IIA had no significant effects on p27 mRNA in pulmonary arteries in HPH rats. Figure 9 showed that hypoxia for four weeks significantly increased Skp2 protein and mRNA level in pulmonary arteries from HPH rats, tanshinone IIA (10 mg/kg/day) significantly reversed the effects of hypoxia on Skp2. So tanshinone IIA regulated Skp2/p27 axis in pulmonary arteries in HPH rats.

## Discussion

In the present study, we demonstrated that tanshinone IIA inhibited the hypoxia-induced PASMCs proliferation by arresting PASMCs in G1/G0-phase. Further, we firstly showed that in vitro tanshinone IIA slowed down the degradation of p27 protein in PASMCs under hypoxic condition via Skp2/Akt-associated pathway and in vivo tanshinone IIA regulated Skip2/p27 axis in HPH rats. This novel information partially clarified the mechanism how tanshinone IIA ameliorated the hypoxia-induced pulmonary arterial remodeling in HPH rats [15].

HPH is associated with persistent increased pulmonary artery constriction and remodeling. Although we have seen advances in the understanding of disease development and treatment, there still lack the ideal therapies for HPH, especially one that can improve the long-term survival of patient with fewer side effects. Tanshinone IIA is one of the pharmacologically active component from Salvia miltiorrhiza, a traditional medicinal herb used for thousands of years in China. Now tanshinone IIA has been widely used in clinic in Asian countries especially in China for the prevention and treatment of cardiovascular diseases without obvious side effects [11], [16]. Previously we found that tanshinone IIA ameliorated HPH in rats [12]. Recently, Wang et al showed that tanshinone IIA prevented both hypoxia and monocrotaline-induced pulmonary hypertension development [17]. The potent dilation on the remodeled pulmonary artery and the inhibition of pulmonary artery remodeling may contribute to the protective effects of tanshinone IIA on HPH [12], [13]. The dilation property of tanshinone IIA on remodeled pulmonary artery may be primarily due to regulating intracellular Ca^2+^ concentration [13] [17]. However, the mechanism underlying anti-remodeling of tanshinone IIA in HPH is not well documented.

Tanshinone IIA is shown to inhibit or modulate the growth, proliferation, migration and apoptosis of diverse cells including keratinocyte, cardiac fibroblast, cardiomyocyte, tumor cells and human aortic smooth muscle cell [18], [19], [20], [21], [22], [23], [24], [25], [26], [27]. Whereas, the hypoxia-induced aberrant proliferation of PASMCs is one of the major causes for pulmonary arterial remodeling. So we hypothesized that inhibition of hypoxia-induce PASMCs proliferation may contribute to its anti-remodeling effects in HPH rats. The present study demonstrated that tanshinone IIA at 3, 10 µg/mL significantly inhibited the hypoxia-induced PASMCs proliferation without obvious effects on PASMCs under normoxic condition.

The anti-proliferation properties of tanshinone IIA on PASMCs may be attributed to its blockade on cell cycle transition. Previous studies have showed that tanshinone IIA arrested cells in S-phase, G2/M-phase or G1/G0-phase dependent on the cell types [20], [22], [28], [29], [30]. So in this study we measured the effects of hypoxia and tanshinone IIA on the cell cycle of PASMCs. The results showed that hypoxia induced more PASMCs to enter G2/S-phase, decreased the cell number in G0/G1-phase. Tanshinone IIA significantly reversed the effects of hypoxia and arrested more PASMCs in G0/G1-phase (figure 2).

Cell cycle is tightly controlled by cyclin-dependent kinases (CDK) and CDK inhibitors, and has been a critical therapeutic target in vascular proliferation-associated diseases. As one of the important CDK inhibitors, p27 has been intensively explored for its potential inhibition on cell cycle in cancer and vascular diseases. Nabel et al found that p27 is one of potent inhibitors of vascular smooth muscle cell growth in vitro and in vivo [31], [32]. Fouty et al demonstrated that p27 modulated PASMCs proliferation during mitogenic stimulation, and over-expression of p27 decreased PASMCs proliferation [6]. Yu et al observed that hypoxia exposure only decreased p27 but not p21 expression in lung in mice, and p27 is the only CDK inhibitor required for the inhibition of PASMCs proliferation and hypoxia-induced pulmonary hypertension by heparin [4], [14]. Here, we found that hypoxia reduced the level of p27 protein but not the level of p27 mRNA in PASMCs. Tanshinone IIA almost reversed the hypoxia-induced reduction of p27 protein. Furthermore, the knockdown of p27 with specific siRNA abolished the effects of tanshinone IIA on p27 protein and PASMCs proliferation, confirming the key role of p27 in anti-proliferation and cell cycle modulation properties of tanshinone IIA.

The expression of p27 was mainly regulated post-translationally, especially at the protein degradation step [8], [33], [34]. In the present study, the degradation assay showed that tanshinone IIA slowed down the degradation of p27 protein and prolonged its half-life from 4.2 h to 15.6 h, which implied that tanshinone IIA also post-translationally regulated p27 expression via inhibiting the degradation.

Skp2 is responsible for the ubiquitin-dependent degradation of p27 protein. It can specifically recognize Thr187-phosphorylated p27 protein and then promote its degradation in nucleus [35], [36]. In many cancers, the increased expression of Skp2 is usually associated with the reduced expression of p27 [37], [38]. Wu et al demonstrated that Skp2 alone is sufficient to promote p27 down-regulation and vascular smooth muscle cell proliferation [39]. However, the mechanisms by which Skp2 activity is regulated remain unknown. Earlier studies revealed that Akt plays a pivotal role in regulating Skp2 and p27. Akt can modulate the transcription, translation and stability of Skp2 then that of p27 through direct and indirect manner [40], [41], [42], [43]. Activation of Akt by diverse extracellular signals triggers cell cascade responses, including cell growth, proliferation, survival, and motility. The level of phosphorylated Akt was showed to be higher in PASMCs in hypoxia [44]. In the present study, we found tanshinone IIA inhibited the hypoxia-induced increase of Skp2 at both mRNA and protein levels. Moreover, the hypoxia-induced increase of phosphorylated Akt also was reduced by tanshinone IIA. These results suggested that tanshinone IIA may protect p27 against being degraded under hypoxic condition at least partially via Akt/Skp2-associated signaling pathway.

An intriguing point is that although under hypoxic condition tanshinone IIA significantly inhibited PASMCs proliferation and regulated the cell cycle, under normoxic condition it almost had no effect on PASMCs proliferation and the cell cycle at low concentration (<30 µg/mL). The specific effects of tanshinone IIA at low concentration on PASMCs under hypoxic condition and its underlying mechanism are needed to explore in future work. Further, whether tanshinone IIA has similar effects on human primary PASMCs proliferation and its effects on other identified pathways regulating cell cycle progression such as p21 and PTEN will be an interesting observation.

**Figure 10:**
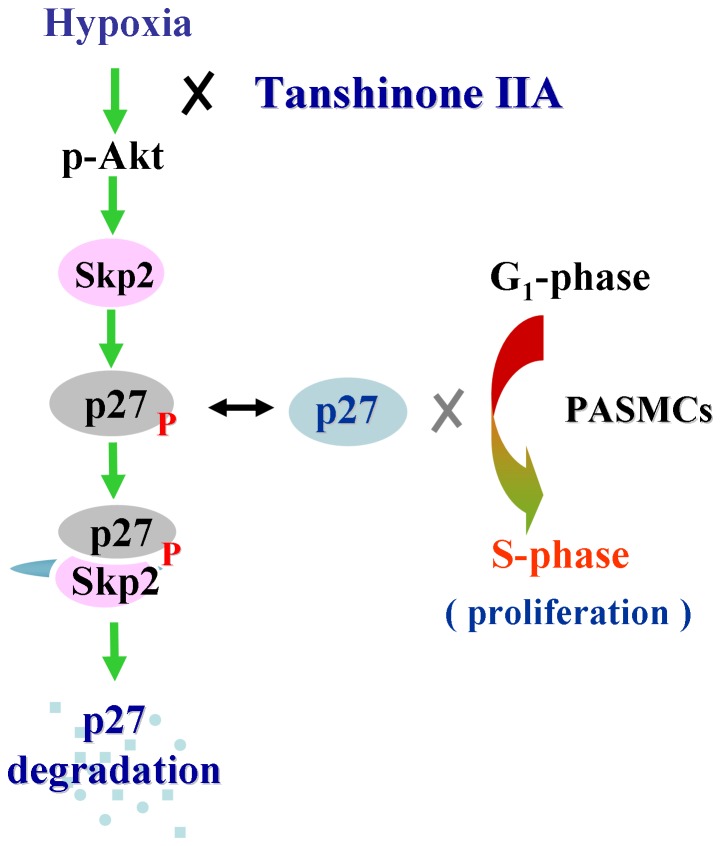
Proposed schematic pathway underlying the inhibition of tanshinone IIA on the hypoxia-induced PASMCs proliferation. P27 blocks the transition of G1-phase to S-phase and inhibits the proliferation of PASMCs. Hypoxia induces the phosphorylation of Akt and the expression of Skp2. Increase of Skp2 specifically promoted the phosphorylation and degradation of p27 protein. So hypoxia promotes PASMCs proliferation via decreasing the protein level of p27. Tanshinone IIA suppresses the hypoxia-induced phosphorylaiton of Akt and the expression of Skp2, further slows down the degradation of p27 protein. So tanshinone IIA may inhibit hypoxia-induced PASMCs proliferation via Akt/Skp2/p27-associated pathway.

In conclusion, we firstly showed that tanshinone IIA inhibited the hypoxia-induced PASMCs proliferation by arresting PASMCs in G1/G0-phase. The effects of tanshinone IIA on PASMCs proliferation and cell cycle was at least due to its slowing down the hypoxia-induced degradation of p27 protein via decreasing the phosphorylation of Akt then the production of Skp2 (Figure 10). These results partially explained the anti-remodeling property of tanshinone IIA on pulmonary artery in HPH. Considering its potent dilative effects on remodeled pulmonary artery form HPH rats [13], the present study suggested that tanshinone IIA, a widely used drug with a well known safety profile, may serve as a new specific and attractive therapy for HPH.
