# Correction: Tanshinone IIA Inhibits Hypoxia-Induced Pulmonary Artery Smooth Muscle Cell Proliferation via Akt/Skp2/p27-Associated Pathway

**DOI:** 10.1371/journal.pone.0108753

**Published:** 2014-09-18

**Authors:** The PLOS ONE Staff

There are multiple errors in the article. Please see below for a description of the errors and their corrections.

In the Funding section, the grant number from the funder National Natural Science Foundation is listed incorrectly. The correct grant number is: 81201516.

There are 2 errors in the author affiliations.

There is an error in affiliation 1 for author Ying Luo. Affiliation 1 should be: Department of Pathophysiology and High Altitude Physiology, College of High Altitude Military Medicine, Third Military Medical University, Chongqing, People’s Republic of China.

The affiliation for the eighth author is incorrect. Zhi-Chao Li is only affiliated with #2 Department of Pathology and Pathophysiology, Fourth Military Medical University, Xi’an, People’s Republic of China.

Dun-Quan Xu and Hai-Ying Dong should not have been attributed equal contribution to this work.

There are a number of errors in the legend for Figure 9, “Effects of Tanshinone IIA on the expression of Skp2 in pulmonary arteries in HPH rats.” The complete, correct Figure 9 legend is: Representative western blot for Skp2 protein (A) and agarose gel electrophoresis for RT-PCR products of Skp2 mRNA (B) in pulmonary arteries from control and HPH rats.

**Figure 9:**
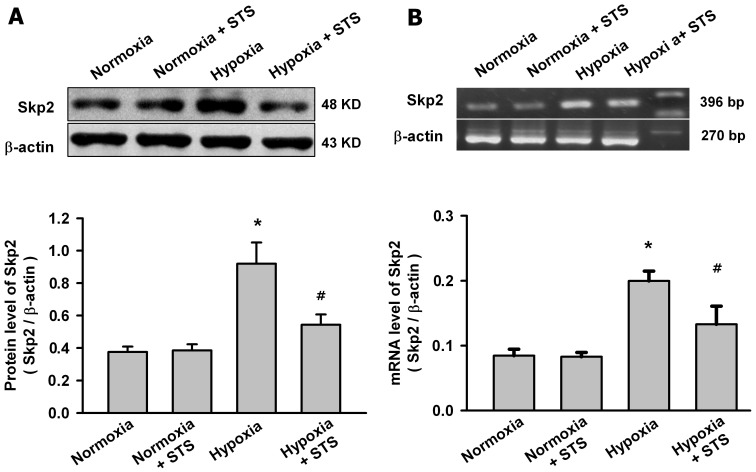
Effects of Tanshinone IIA on the expression of Skp2 in pulmonary arteries in HPH rats. Representative western blot for Skp2 protein (A) and agarose gel electrophoresis for RT-PCR products of Skp2 mRNA (B) in pulmonary arteries from control and HPH rats. Beta-actin was used as control. Summarized data are shown in the bottom respectively. Hypoxia significantly increased the Skp2 protein and mRNA level in pulmonary arteries from HPH rats, tanshinone IIA significantly reversed the effects of hypoxia on the expression of Skp2 (*P<0.05 vs nomoxia group, # P<0.05 vs hypoxia group, mean ± SEM, n  =  3).
